# Improved emotional conflict control triggered by the processing priority of negative emotion

**DOI:** 10.1038/srep24302

**Published:** 2016-04-18

**Authors:** Qian Yang, Xiangpeng Wang, Shouhang Yin, Xiaoyue Zhao, Jinfeng Tan, Antao Chen

**Affiliations:** 1 Key Laboratory of Cognition and Personality of Ministry of Education, Faculty of Psychology, Southwest University, Chong, Qing, China

## Abstract

The prefrontal cortex is responsible for emotional conflict resolution, and this control mechanism is affected by the emotional valence of distracting stimuli. In the present study, we investigated effects of negative and positive stimuli on emotional conflict control using a face-word Stroop task in combination with functional brain imaging. Emotional conflict was absent in the negative face context, in accordance with the null activation observed in areas regarding emotional face processing (fusiform face area, middle temporal/occipital gyrus). Importantly, these visual areas negatively coupled with the dorsolateral prefrontal cortex (DLPFC). However, the significant emotional conflict was observed in the positive face context, this effect was accompanied by activation in areas associated with emotional face processing, and the default mode network (DMN), here, DLPFC mainly negatively coupled with DMN, rather than visual areas. These results suggested that the conflict control mechanism exerted differently between negative faces and positive faces, it implemented more efficiently in the negative face condition, whereas it is more devoted to inhibiting internal interference in the positive face condition. This study thus provides a plausible mechanism of emotional conflict resolution that the rapid pathway for negative emotion processing efficiently triggers control mechanisms to preventively resolve emotional conflict.

Emotional conflict refers to the interference of goal-irrelevant emotional stimuli on goal-relevant emotional stimuli^1^, which normally needs to be suppressed through conflict control mechanisms to optimise goal-directed behaviour^2^,^3^. In the laboratory, the effect of emotional conflict is frequently observed in the emotional face-word Stroop task, where participants are required to respond to the emotional words that may be congruent (congruent trial, C), or incongruent (incongruent trial, I) with the contextual emotional faces^4^,^5^,^6^. With regard to neural mechanisms underpinning emotional conflict processing, even though evidences from emotional conflict adaptation have suggested the involvement of the rostral anterior cingulate cortex (rACC) in conflict resolution^7^,^8^, there is still evidence to highlight the engagement of the prefrontal cortex in this process, such as the lateral prefrontal cortex (LPFC) in interference control process. More specifically, the LPFC has been considered to be involved in integrating cognitive and affective processes^9^,^10^, as well as be implicated in modulating top-down and bottom-up neural circuits that underlie emotional conflict control^6^,^11^,^12^.

Previous studies have proved specific effects of emotion on cognitive processes, spanning from perceptual processing to cognitive control, of which emotions are normally reflected in negative stimuli, or emotions are not distinguished between negative and positive. However, it has been argued that positive and negative emotions actually have different effects on mental processes^13^,^14^. In general, negative stimuli receive processing priority^14^; as such, they are processed faster and can therefore improve the behavioural performance in cognitive contexts through more processing resources devoted to emotional-related target processing^3^,^15^,^16^,^17^. For example, negative distracters could facilitate cognitive conflict resolution, which was achieved through earlier conflict modulation elicited by affective information^15^. This effect has been confirmed by evidences from neuroimaging studies, which showed that negative emotion elicited the enhancement of the control in LPFC^18^,^19^. With regard to the neural substrates that underlie the processing priority of emotional stimuli, the pulvinar and the amygdala are thought to be core regions implicated in evaluating the emotional significance of a stimulus^20^. In particular, the two areas are involved in responding to sources of potential threat in a rapid and automatic way, which has been demonstrated by neurobiological and neuroimaging studies^21^,^22^. More importantly, affective information processed in the amygdala, as well as the pulvinar could be directly conveyed to control structures, such as the LPFC, enabling them to implement goal-directed control function^20^.

Based on previous studies, it is reasonable to assume that the processing priority of emotional information might affect the resolution of emotional conflict, as shown through different behavioural and neural patterns for negative and positive emotional contexts. In light of no study has examined this issue to date, the present study aimed to clarify the effect of emotional processing priority on emotional conflict control, as well as the underlying neural mechanisms. To this end, we adopted a typical emotional face-word Stroop task manipulating the valence of the distracting face, which allowed us to test the potential effect of the processing of distracting emotional stimuli on emotional conflict control, given that a significant effect of valence can be elicited by emotional faces, rather than by emotional words^23^. At the neural level, we initially investigated regional activation patterns under different experimental conditions. In light of the LPFC is a key region involved in emotional conflict control, we defined it as a seed region to explore the functional connectivity patterns for the negative face condition and the positive face condition, respectively, which was calculated using psychophysiological interaction (PPI) analysis. We hypothesized that the emotional conflict effect would be differentially affected by the valence of emotional face; likewise, this difference would be accompanied by distinct regional activation patterns. In addition, we expected that the LPFC would exhibit different functional connectivity patterns for the negative and positive emotional contexts, showing that valence-dependent control functions can be ascribed to the processing priority of emotional information.

## Results

### Behavioural data

**Figure 1:**
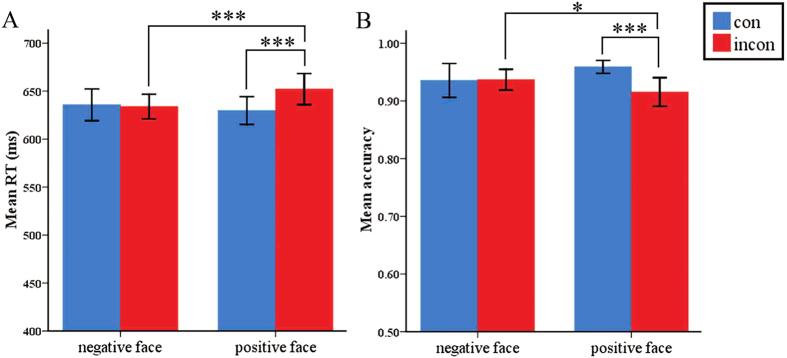
Behavioural results. (**A)** The average reaction time (RT) for four conditions. **(B)** The accuracy for four conditions. *P < 0.05, ***P < 0.001.

A 2 (emotional congruency: congruent or incongruent)
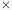
2 (the valence of emotional face: negative or positive) repeated-measures ANOVA was conducted on reaction time (RT) and accuracy. The statistical results for RT are displayed in Fig. 1A. There was a significant main effect of congruency (*F*_1, 30_ = 33.719, *P* < 0.001) (incongruent: 642 ms vs. congruent: 632 ms), a significant main effect of emotional face valence (*F*_1,30_ = 12.556, *P* = 0.001) (positive face: 640 ms vs. negative face: 634 ms), and a significant interaction effect (*F*_1,30_ = 10.719, *P* = 0.003). Follow-up analyses revealed that the conflict effect was absent for negative faces (*F*_1,30_ = 0.165, *P* = 0.687); in contrast, for positive faces, responses for congruent trials were faster than those for incongruent trials (*F*_1,30_ = 32.970, *P* < 0.001). In brief, the congruency effect was different between the contexts of positive and negative distracting faces.

The accuracy results are illustrated in Fig. 1B. The main effect of congruency was significant (*F*_1,30_ = 17.274, *P* < 0.001), but the main effect of valence of emotional face was not significant (*F*_1,30_ = 0.033, *P* = 0.857). The interaction effect was significant (*F*_1,30_ = 5.873, *P* < 0.022). Follow-up analyses showed that in the negative face condition, there was no significant difference between congruent and incongruent (*F*_1,30_ = 0.014, *P* = 0.908), whereas in the positive face condition, the accuracy for congruent trials was significantly higher than that for congruent (*F*_1,30_ = 18.902, *P* < 0.001).

### Regional brain activation

**Figure 2:**
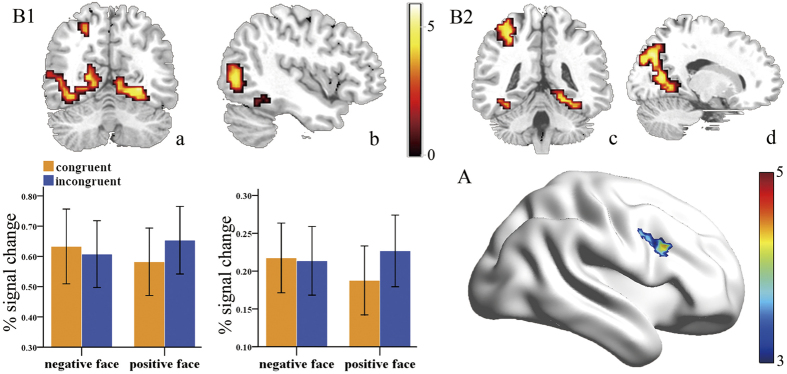
(**A)** Regional brain activation for the congruency effect: DLPFC. (**B)** Regional brain activation for the contrast of [(ip-cp)-(in-cn)]. (**B1)** Significant activation was found in areas associated with emotional face processing (FFA, MOG, MTG). a: fusiform gyrus (FFA), b: middle occipital gyrus (MOG), middle temporal gyrus (MTG). Specific activity patterns across four experimental conditions for these areas were showed in the bottom panel. (**B2)** Significant activation was found in DMN (PCC/precuneus), IPL. c: inferior parietal lobule (IPL), d: precuneus/posterior cingulate gyrus (PCC).

For the congruency effect, the activation of several voxles located in the right DLPFC (x = 48, y = 18, z = 27) (P < 0.05 SVC corrected) was observed; those voxels showed higher activation for incongruent trails compared with congruent trials. This result is displayed in Fig. 2A. For the effect of emotional face valence, no voxels showed significant activation for the contrast of negative minus positive at the level of *P* < 0.05, FWE corrected. The Paired t-test for the contrast of (P (i − c)−N (i − c)) resulted in several regions, including the posterior cingulate cortex (PCC) (−15, −75, 24), precuneus (−18, −72, 33), inferior parietal lobule (IPL) (−36, −45, 57), the fusiform gyrus (FFA) (30, −60, −12), middle occipital gyrus (MOG) and the middle temporal gyrus (MTG) (42, −75, 0). These regions were remained significant at a significance level of *P* < 0.05, FWE corrected. These results are presented in Fig. 2B1. In addition, activity patterns across four experimental conditions in areas associated with emotional faces processing were showed in Fig. 2B1 (the bottom panel). Other activation during the Paired t-test was found in the Table 1.

### ROI analyses

For the right pulvinar, there was a significant effect of the interaction between the congruency and emotional face valence (*F* = 9.430, *P* = 0.005). No significant main effect of congruency or emotional face valence was detected. The follow-up t-test showed that in the congruent condition, the regional activation for negative faces was significantly higher than that for positive faces (*F* = 4.165, *P* = 0.051).

**Figure 3:**
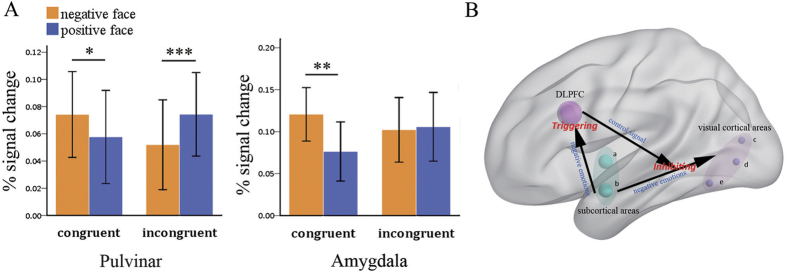
(**A**) Activity intensities across four experimental conditions for ROI analyses, of which ROIs include the pulvinar and the amygdala. **(B)** Plausible emotional conflict mechanism relying on the interaction of emotional information and control signal that recruits subcortical areas [a: the pulvinar, b: the amygdala], visual cortical areas [middle occipital gyrus (MOG), middle temporal gyrus (MTG), fusiform gyrus area (FFA)], and the control area (DLPFC). *P < 0.05, **P < 0.05, ***P < 0.001.

For the right amygdala, there was a significant main effect of emotional face valence (*F* = 6.476, *P* = 0.016), which was driven by higher activities for negative faces compared with positive faces. However, the main effect of congruency was not significant. In addition, the effect of the interaction between congruency and emotional face valence was significant (*F* = 5.214, *P* = 0.03); specifically, activities were higher for the negative faces than for the positive faces in the congruent condition (*F* = 8.93, *P* = 0.006). The results were presented in Fig. 3A.

### Functional connectivity: PPI

**Figure 4:**
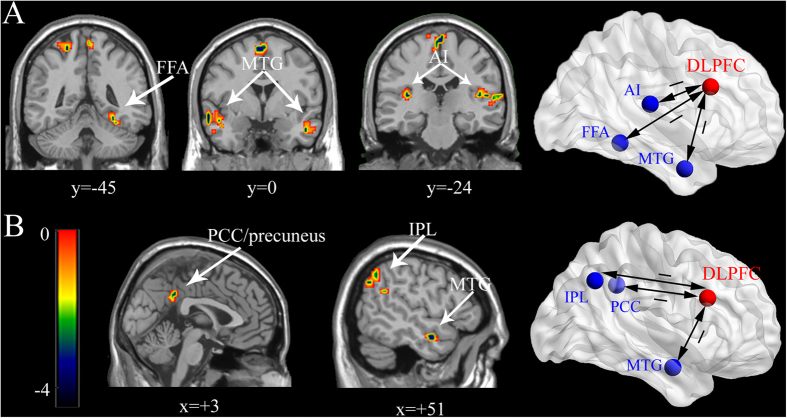
PPI results for negative and positive face contexts. (**A)** In the negative face context: negative coupling patterns between the seed region (DLPFC) and brain regions associated with emotional conflict processing. (**B)** In the positive face context: negative coupling patterns between the seed region (DLPFC) and brain regions in DMN (PCC/precuneus, IPL), as well as MTG.

One-sample t-test of PPI showed that during the negative face condition, the DLPFC negatively connected with the FFA (30, −45, −12), the AI (48, −24, 15), the MTG (48, 0, −30) and the MOG (−45, −75, −12) during the process of conflict control. In contrast, during the positive face condition, the DLPFC negatively coupled with the MTG (54, −6, −21), the PCC/precuneus (3, −48, 30), and the IPL (51, −45, 24). All the regions were remained significant at the level of *P* < 0.005, uncorrected. The results are presented in Fig. 4. In the meanwhile, Fig. 3B illustrated the plausible neural mechanism of emotional conflict control, of which negative emotions rapidly trigger DLPFC through the subcortical route, and then, DLPFC inhibited emotional conflict timely in the visual cortical areas.

## Discussion

To investigate the effect of the emotional valence of distracting faces on emotional conflict control, the current study examined the conflict effect under negative and positive faces, respectively. Behaviourally, emotional conflict was eliminated in the negative face condition, which was driven by the significant reduced reaction time for negative faces compared to positive faces in incongruent trials, whereas the significant conflict effect was observed in the positive face condition. In parallel with the result pattern at the behavioural level, the fMRI data showed the null conflict effect in areas related to emotional face processing (the FFA, MTG, and MOG) for negative faces, whereas the significant conflict effect was found for positive faces. This actually reflected the interaction effect between the valence of emotional face and congruency. Likewise, this interaction effect was observed in the DMN (the IPL, PCC, and precuneus). Interestingly, we found the processing advantage of negative emotions reflected in both subcortical areas (amygdala, pulvinar) and visual cortical areas. Furthermore, at the functional connectivity level, the DLPFC negatively coupled with those areas implicated in emotional processing (the FFA, MTG, MOG, and AI) in the negative face context, whereas negatively coupled with DMN (the PCC/precuneus, and IPL) in the positive face context. The distinct connectivity patterns between negative faces and positive faces might be driven by the primacy processing of negative faces, given that the rapid extraction of negative emotion is able to affect cortical control processes spanning from perceptual phase to cognitive control. Taken together, neuroimaging results indicated that the lack of conflict effect for negative faces is possibly ascribed to the more efficient exertion of conflict control induced by the processing priority of negative faces.

It is plausible to investigate neural mechanisms of emotional conflict processing in the positive face context because of the occurrence of conflict effect. It has been suggested that the cognitive conflict normally stems from two aspects: internal noise and external distracters^24^. Of note, the interaction effect between emotion and congruency in the current data was observed in areas related to emotional face processing (the FFA, MTG, MOG), as well as the DMN (the precuneus, PCC, and IPL), suggesting that the generation of emotional conflict, which is similar with the cognitive conflict, has external and internal sources as well. More specifically, the FFA, MTG and MOG were involved in emotional faces processing, which acted as a source of interference from external input; this result is consistent with previous findings that emotional faces could activate ventral temporal occipital visual areas^25^,^26^, confirming the view of the processing of emotional faces occurs in the perceptual phase, even though they are task-irrelevant^27^. Furthermore, the external interference due to incompatible emotional valences between words and faces occurs at relatively early stages, given that the activation of emotional faces processing during perceptual phases results from emotional evaluation of the stimuli, rather than simply relating to particular visual features^28^,^29^. On the other hand, spontaneous cognitive activities, such as mind wandering, are thought to be a source of internal interference^30^, which involves activation of the DMN^31^. The result of increased activities in the DMN (the PCC, precuneus, and IPL) in the current study thus supports the view of internal noises also serve as sources of emotional conflict^30^,^32^, simultaneously in line with the finding that the intrusion of mind wandering occurs more frequently in the positive face condition^33^.

In parallel with the absent emotional conflict for negative faces at the behavioural level, regional activations for emotional face processing areas (the FFA, MTG, and MOG) did not show a congruency effect in the negative face condition as well. One possibility for this effect is that negative faces do not interfere with primary task; alternatively, the emotional conflict actually emerges, but it is totally resolved. One argument can be put forward to refute the former possibility. Compared with positive faces, negative faces are more readily to be processed^34^, implying that larger interference is elicited by negative distracters relative to positive distracters. In light of the significant conflict effect observed for positive faces in the current data, the conflict effect should be appeared (even larger than that for positive faces) in the negative face condition as well; however, this result didn’t show in the current study. It is therefore reasonable to speculate that the lack of emotional conflict for negative faces is possibly due to the timely conflict resolution implemented by the control mechanism, rather than negative faces do not interfere with the primary task. Besides the existing viewpoint from empirical research, present results support the speculation regarding the reason for the lack of conflict effect through activity patterns observed in areas implicated in emotional face processing in the congruent condition. Specifically, activities in subcortical areas (amygdala, pulvinar), as well as temporal-occipital cortical areas (the FFA, MTG, and MOG) were higher in the negative face condition relative to the positive face condition; likewise, a significant effect of negative faces observed in the amygdala. Obviously, in the condition of the emotional valence between words and faces is consistent, it is less likely to suppress emotional faces processing as it elicits fewer destructive effects on goal-related task. Furthermore, the two subcortical areas (amygdala, pulvinar) are devoted to prioritizing the processing of emotional stimuli^35^, especially for negative emotions, indicating an “emotional prioritization” can be reflected even under unattended conditions.

It has been suggested that the conflict effect is induced by the processing of distracters. However, it is notable that emotional distracters do not always induce interference; instead, they can improve, rather than weaken task performance, possibly because of the emotional prioritization. At the behavioural level, emotional distracters have various effects on behaviour performance, spanning from perceptual processing to cognitive control, of which includes conflict processing^36^,^37^. At the neural level, the ability to detect potential threats involves specialized neural systems that can promote rapid response to allow adaptive behavior. A further example is given by the early timing of amygdala-ACC activations (at 100 ms), suggesting that fast reactions to a potential threat is facilitated by a specialized frontal-limbic network, which to a certain extent reflects the interactive process of emotion and conflict processing. With regard to the beneficial emotional modulation on cognitive conflict processing, the ERP study showed that decreased conflict effects occur when distracting emotional information is integrated with the target stimulus, as subliminal presentation of emotional words modifies conflict processing at a relatively early stage (190 ms post-stimulus onset)^16^. In the meanwhile, the fMRI study showed that conflict effects reduce and emotion speeds up conflict processing when negative emotion acted as distracting information^17^. In brief, the fast modulatory influence of negative emotion on conflict processing ensures rapid resolution of conflict in potentially threatening situations^3^,^16^. Despite this facilitatory effect of distracting emotion on conflict processing was observed in the cognitive domain; however, these consistent results regarding the effect of emotional prioritization on conflict processing raises a possibility that this emotional effect also be suited to the emotional domain, despite the specific modulatory mechanism might be different.

The present results indicated that the processing priority of negative faces is essentially reflected in the pulvinar and the amygdala. More importantly, the pulvinar has been demonstrated to show synchronous activity with multiple cortical areas^38^. Neuroanatomically, connections between the amygdala, the pulvinar and the PFC^20^,^38^,^39^,^40^ are thought to process fear-related stimuli rapidly and non-consciously^41^. In this case, it is conceivable that the efficient conflict control process can be rapidly triggered by distracting negative emotions through the subcortical pathway, which in turn help to resolve conflict. Actually, this mechanism is highly adaptive as emotional stimuli signal the particular relevance of a situation that may require especially efficient attentional control. In accordance with this view, the functional connectivity results supply further evidences for the underlying neural mechanisms of how optimal control is implemented when it has been initiated by negative emotions so as to resolve conflict. In the negative face condition, the DLPFC negatively coupled with regions associated with emotional face processing, including the FFA, MTG, MOG and AI^42^,^43^. These negative couplings thus suggest that the cognitive control area (DLPFC) could function to suppress the processing of distracting negative faces in visual areas, and therefore eliminating emotional conflict by blocking resources available to distracters processing. This is in line with a previous finding that the DLPFC was not only responsible for resolving cognitive conflict, but also was engaged in reducing the impairment of emotional stimuli^44^. What’s more, this modulatroy effect of emotions on conflict control observed in visual regions might reflect the fact that regional competition between the two information flows of the target and the distracter occurred during the perceptual process^45^,^46^. Ultimately, this competition can be stopped timely by attenuating distracters processing.

Despite the negative face acts as the distracter in our study, and is even characterized by significant processing priorities; however, with evidences from this study, it is hypothesized that the negative face might serve as a contributing factor that triggers the PFC through the faster emotional processing pathway centered on the pulvinar and amygdala to implement supervisory control, which in turn inhibits or blocks the processing of the distracting emotional face through a cortical pathway^47^. This plausible pathway is also evidenced by the claim that emotional information can be rapidly conveyed to visual cortical areas through the subcortical emotional processing pathway^48^. For example, rapid initial processing of negative (fear) stimuli occurs at around 100 ms, whereas emotional effect reflected in visual cortical areas occurs at 150–200 ms. To some extent, this mechanism of supervisory emotional conflict control essentially results from the combination effect of emotion and control process.

The rapid control mechanism might benefit from the processing advantage of negative faces; however, the processing priority of positive emotions is normally inferior to negative emotions, and this distinction is independent of attention^14^. It is therefore that positive faces might fail to timely trigger regulatory mechanisms to resolve conflict. Instead, as we discussed regarding the occurrence of emotional conflict above, besides the interference elicited by positive faces, mind wandering interferes with the goal-directed task as well. This internal interference not only impairs the emotional control ability, but also increases automatically elicited task-unrelated thoughts^49^. As the results showed that, the DLPFC had negative couplings with regions pertaining to the DMN (the PCC/precuneus, and IPL) in this condition; this observation reflects that conflict control is engaged in the suppression of internal distraction^50^, which weakens the functioning of control for positive face inhibition. This idea is consistent with a previous finding that mind wandering is associated with cognitive control recruitment^51^. On the other hand, it has been suggested that positive emotions could broaden the scope of attention^52^, and as a result, unattended information, such as the positive face, is more likely to be fully processed^53^. According to the dual-competition model^16^, as well as the view about the integration of emotion and cognitive control, emotional processing is also concerned with the DLPFC^54^. Taken together, the increased sources of distraction in the internally and externally driven streams competed for shared processing resources for conflict control, and as a consequence, it was difficult for positive faces (external distraction) to be completely inhibited through control mechanisms due to the limited control capacity^51^.

To conclude, the main goal of the present study was to investigate the effect of positive and negative distracting stimuli on emotional conflict control, as well as the underlying neural mechanisms. Consistent with our assumption, emotional conflict was absent in the negative face condition, more importantly, the underlying neural mechanism of this effect was that the prioritization of negative emotions facilitates emotional conflict resolution. Specifically, the conflict control mechanism can be efficiently triggered by rapid emotional processing to implement a supervisory control, which in turn inhibits or blocks the processing of the distracting emotional face through a cortical pathway. This mechanism may provide a possible account for the elimination of emotional conflict in the negative face condition. In the meanwhile, the present findings highlight the engagement of the DLPFC in modulating emotional conflict control, and also, enrich the deeper understanding of the emotional processing priority effect on conflict control from the cognitive domain to emotional domain.

## Methods

### Participants

Thirty-five right-handed students from Southwest University in China participated in this experiment. Participants were excluded if their head motion is larger than 2.5 mm and 2.5 degree, as a result, 31 participants (19 males, 12 females, mean age = 22 ± 1.8, ranged from 18 to 25) included in the final analyses. All participants had normal or corrected-to-normal vision and reported no history of neurological disease. The university Human Ethics Committee for the Brain Mapping Research approved this study. The methods were carried out in accordance with the approved guidelines. All participants gave written informed consents and were paid for participating.

### Stimuli and experimental procedure

We used an emotional face-word Stroop task to elicit emotional conflict. The emotional faces were placed in the centre of a screen in that was 4° wide 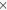
5° high. The photograph of happy and fearful facial expressions included 10 males and 10 females that were selected from the native Chinese Facial Affective Picture System (CFAPS)^55^. The arousal evoked by the positive and negative faces did not significantly differ (*t* = 1.12, *P* = 0.28). The Chinese words “yukuai” (which means happy) and “kongju” (which means fear) were written in red superimposed over the middle of the emotional faces, almost at the location of the nose; the Chinese words were approximately 2.5° wide 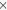
1.3° high. Consequently, the valence of the emotional word was either congruent or incongruent with the valence of the emotional face. Task presentation and behavioural responses recording were performed using the E-Prime software.

We specifically focused on the congruency effect for the emotional valence of the distracting face (negative face [incongruent – congruent] and positive face [incongruent – congruent]) based on the assumption that differences in reaction mainly stem from the effect of the valence of the emotional face. Therefore, the experimental task consisted of 2 × 2 conditions that varied according to emotional congruency (congruent or incongruent), and the valence of the emotional face (positive or negative). The present study employed an event-related design and consisted of 4 runs of 80 trials, with an equal proportion of each condition. The stimuli were presented for 1000 ms, with a varying inter-stimulus interval (ISI) of 3000–5000 ms (mean ISI = 4000 ms) during which a central fixation point was shown. Stimuli were presented in a pseudorandom order (counterbalanced for equal numbers of congruent and incongruent stimuli per run, and the gender of the emotional faces were also counterbalanced across trial types). There was no immediate repetition of the same face. The participants were asked to judge the valence of the emotional words as quickly and as accurately as possible by pressing the button “1” (right index finger) for happy and “2” (right middle finger) for fearful.

### Behavioral data analyses

The behavioral data were analysed using SPSS, the error trials and trials with RTs larger than 3 SDs from the mean were excluded from the RT analyses. We estimated the main effect of the congruency, the valence of emotional face, and their interaction effect on reaction time (RT) and accuracy.

### fMRI data analyses

#### MR image acquisition

Images were acquired with a Siemens Trio 3.0T scanner in the Laboratory of Cognition and Personality in Southwest University, China. A T2-weighted echo-planar imaging (EPI) sequence was used for functional data collection (TR = 1500 ms; TE = 30 ms; flip angel = 90°; acquisition matrix = 64 × 64; 24 interleaved 5 mm-thick slices; inter-slice skip = 1 mm). At the end of the experiment, a total of 176 T1-weighted images were recorded with a thickness of 1mm and in-plane resolution of 0.98 × 0.98 mm^2^ (TR = 1900 ms; TE = 2.52 ms; flip angel = 90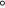; acquisition matrix = 256 × 256).

#### Pre-processing

All images were analysed using SPM8 (the Welcome Trust Centre for Neuroimaging, University College London, UK; http://www.fil.ion.ucl.ac.uk/spm/software/spm8/). Statistical analyses were preceded by the following pre-processing steps: the first six scans of each run were discarded to allow for signal stability following onset transients. Slice timing was used to correct slice order. To reduce the effect of head motion, the data were realigned to the first scan of each run, and participants whose head movements exceeded 2.5 mm and 2.5° were excluded. The images were then normalized to MNI space in 3 × 3 × 3 mm^3^ –sized voxel. The normalized data were spatially smoothed with a Gaussian kernel, the full width at half maximum (FWHM) of which was specified as 6 × 6 × 6 mm^3^.

#### Whole-brain analyses

To detect the task-related regional activations, we employed a general linear model (GLM) in SPM8. The model incorporated four regressors: congruent-negative face (cn), congruent-positive face (cp), incongruent-negative face (in), and incongruent-positive face (ip), with six realignment parameters included as covariates. All of the regressors were convolved with the canonical hemodynamic response function (HRF). At the individual level, we created a contrast of incongruent vs. congruent to detect brain regions responsible for conflict control, a contrast of negative face vs. positive face to detect regional activation related to emotional face valence processing, and two contrasts of incongruent vs. congruent for the negative face and positive face condition to examine the emotional faces-dependent effects of conflict control. At the group level, separate one-sample t-tests were performed to explore the main effects of congruency and emotional face valence. In addition, a paired t-test was performed to explore differences between the contrasts ip-cp and in-cn. To further visualize neural activation patterns for each of four experimental conditions, percent signal change were extracted from clusters showing significant interaction effects, particularly in areas associated with emotional faces processing. Because conflict control was hypothesized to be related to the regional activation in the PFC, so the congruency effect for the PFC was corrected for a small-volume family-wise error (FWE) at the voxel level. The PFC was defined according to the Automated Anatomical Labeling software implemented in the WFU PickAtlas^56^. For other effects of interest, the regional activation was corrected for a FWE threshold of *P* < 0.05 at the whole - brain voxel level.

#### ROI analyses

The ROI analyses were performed within two previously defined seed regions: the pulvinar (12, −2, 0) and the amygdala (26, −2, −16)^43^. Spheres with a 6 mm radius were positioned at the local peak. The two regions have been considered as critical for the implicit processing of emotional stimuli, especially for negative emotions. The percent signal change was extracted from the regions for each condition and then repeated-measures ANOVA and follow-up analyses were performed to examine the effects of interest^57^, which mainly aims to confirm processing priority of negative faces.

#### Functional connectivity analyses

PPI analyses were used to assess context-dependent variations in the functional connectivity. PPI is a regression-based method for functional connectivity that examines changes in the regression slope of activation between a seed region and other regions under the influence of different experimental contexts^58^.

PPI analyses in the present study were performed in an attempt to clarify the functional connectivity patterns during conflict control under the positive and negative face contexts, respectively. Based on the current result for the congruency effect, as well as previous findings regarding emotional conflict control, we defined the DLPFC as a region of interest. The VOI time course (the physiological variable) was extracted from the seed region (x = 48, y = 18, z = 27) (6 mm radius sphere at the local peak). The psychological variables in the study were defined as N (i minus c) and P (i minus c) for the negative and positive face condition, respectively. PPI analyses here were performed to explore brain regions showing significant connectivity with the DLPFC. Afterwards, separate one-sample t-tests were conducted to detect the group-level functional connectivity patterns for negative and positive faces.

## Additional Information

**How to cite this article**: Yang, Q. *et al.* Improved emotional conflict control triggered by the processing priority of negative emotion. *Sci. Rep.* **6**, 24302; doi: 10.1038/srep24302 (2016).

## Figures and Tables

**Table 1:** Brain regions associated with significant BOLD signal increases for the distinct congruency effect between positive faces and negative faces.

Region	Hemisphere	Peak t-value	Voxels	MNI Coordinates		
				x	y	z
[(ip-cp)-(in-cn)]						
Precuneus	L^a^	5.78	143	−18	−72	33
Fusiform Gyrus	R^a^	5.39	73	30	−60	−12
Posterior Cingulate	L^a^	5.25	32	−15	−75	24
Middle Occipital Gyrus	R^a^	5.20	54	42	−75	0
Inferior Parietal Lobule	L^a^	4.90	109	−36	−45	57
Lingual Gyurs	R^a^	4.85	101	18	−57	−9
Precentral Gyrus	L^a^	4.75	109	−39	−12	60
insula	L^b^	4.02	13	−33	−15	15
Middle Temporal Gyrus	R^b^	3.92	16	48	−78	12

^a^FWE threshold of *P* < 0.05 at the whole brain level.

^b^FDR threshold of *P* < 0.05 at the whole brain level.
